# Optimal neural inference of stimulus intensities

**DOI:** 10.1038/s41598-018-28184-5

**Published:** 2018-07-03

**Authors:** Travis Monk, Cristina Savin, Jörg Lücke

**Affiliations:** 1 Neurosensory Science, Cluster of Excellence Hearing4all, University of Oldenburg, Oldenburg, 26129 Germany; 2 Center for Neural Science and Center for Data Science, NYU, New York, 10003 USA

## Abstract

In natural data, the class and intensity of stimuli are correlated. Current machine learning algorithms ignore this ubiquitous statistical property of stimuli, usually by requiring normalized inputs. From a biological perspective, it remains unclear how neural circuits may account for these dependencies in inference and learning. Here, we use a probabilistic framework to model class-specific intensity variations, and we derive approximate inference and online learning rules which reflect common hallmarks of neural computation. Concretely, we show that a neural circuit equipped with specific forms of synaptic and intrinsic plasticity (IP) can learn the class-specific features and intensities of stimuli simultaneously. Our model provides a normative interpretation of IP as a critical part of sensory learning and predicts that neurons can represent nontrivial input statistics in their excitabilities. Computationally, our approach yields improved statistical representations for realistic datasets in the visual and auditory domains. In particular, we demonstrate the utility of the model in estimating the contrastive stress of speech.

## Introduction

The intensity of a sensory stimulus can carry important information. For example, consider the sentence ‘Calvin yelled something at Hobbes’. A speaker can change the meaning of the sentence by stressing certain words in it. ‘Calvin *yelled* something at Hobbes’ emphasizes that Calvin did not speak in a normal voice but that he yelled. ‘Calvin yelled something at *Hobbes*’ emphasizes that Hobbes, and not somebody else, was the recipient of Calvin’s yelling. Stressing other words, or combinations of words, will imply other meanings, a linguistic technique termed contrastive stress^1^. How might neural circuits estimate the contrastive stress of a sentence? More generally, how might neurons learn and represent the intensity of stimuli?

**Figure 1:**
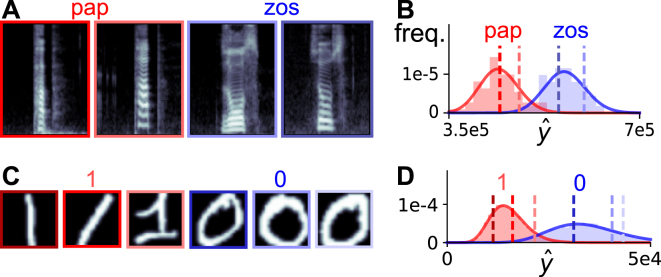
Intensity and class are correlated in natural data. (**A**) Example log-mel spectrograms of logatomes ‘pap’ and ‘zos’ from the raw OLLO dataset. (**B**) Histograms of the brightness $\hat{y}$ (the sum of the spectrogram values) across utterances for each class and corresponding Gamma fit. Brightness values corresponding to examples in A are marked by dashed lines. (**C**) Example images of classes ‘1’ and ‘0’ from the raw MNIST dataset. (**D**) Same as B, in the visual domain.

One naive solution would be to infer that a word is stressed if it is louder than other words, since stress and loudness are often correlated^2–4^. This proposal fails because the intensity of a stimulus (utterance) depends on its class (word). As an illustration, consider the logatomes ‘pap’ and ‘zos’, taken from the raw Oldenburg Logatome Corpus dataset (OLLO)^5^, with example spectrograms shown in Fig. 1A. The total energy in time-frequency space, or their ‘brightness’ $\hat{y}$, differs across individual logatomes. Furthermore, in this dataset we see that there are systematic differences in intensity for the two logatome classes, with ‘zos’ generally being louder than ‘pap’ (Fig. 1B). If stress were determined by a threshold on brightness given by the average intensity across classes, then we would incorrectly label most ‘zos’ logatomes as stressed and most ‘pap’ logatomes as de-stressed. This example reveals a key insight about the statistics of natural stimuli: intensity and class information depend on one another. This observation is not restricted to the auditory domain but holds true across sensory modalities (Fig. 1C). Hence, making correct judgements about intensity needs to consider the stimulus class. Conversely, intensity information needs to be considered when estimating the stimulus class.

Traditional models of sensory processing ignore dependencies between intensity and stimulus class^6,7^. Most models treat stimulus intensity as a nuisance variable that is typically removed by some form of *ad hoc* normalization^8–11^. Such preprocessing discards a potentially useful source of information that could have been used to make better inferences about the stimuli. Beyond being computationally inefficient, these models cannot explain a key feature of sensory perception: information about intensity is consciously accessible. For example, we can detect the stress of utterances, or whether a scene is being viewed at midday or at dusk. In summary, existing models ignore an important statistical feature of natural stimuli. Furthermore, it remains unclear how joint inference of intensity and stimulus class can be achieved by neural circuitry.

Here we use a novel and flexible probabilistic generative model to investigate statistical dependencies between the intensity and class of a stimulus. Our model is a rare instance of a generative model that is analytically tractable, yielding closed-form joint and marginal inference for both stimulus class and intensity. Moreover, we find that these computations can be effectively approximated by biologically-plausible neural plasticity and dynamics. We show that a neural circuit equipped with two specific plasticity rules can learn the statistics of sensory inputs. Hebbian plasticity allows the circuit’s synapses to capture the features of classes, consistent with other unsupervised learning algorithms^8,12–18^. Intrinsic plasticity (IP)^19,20^ adapts the overall excitability of a neuron to reflect the average intensity of each input stimulus class. From a biological perspective, our results provide a principled, normative, and testable computational account for the role of IP in sensory learning. Intensity estimation and IP thus become part of the general approach of perception as probabilistic inference^21–24^. From a machine learning perspective, the derived results provide novel and efficient implementations for a difficult statistical problem, with applications in auditory processing and beyond.

## Results

### Modeling statistical dependencies between stimulus class and intensity

**Figure 2:**
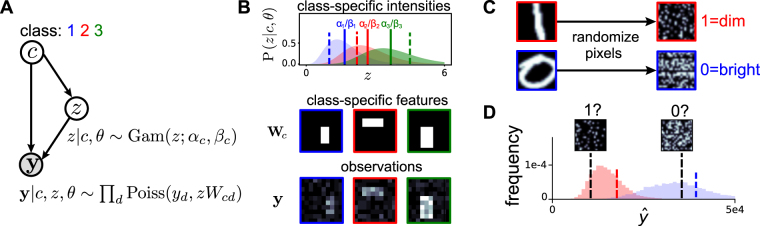
Modeling statistical dependencies between the class and intensity of a stimulus. (**A**) Generative model schematic: given a class *c*, the intensity *z* is drawn from a Gamma distribution, with class-specific parameters; the data point **y** is then generated by adding Poisson noise to the scaled features *z***W**_*c*_. (**B**) A simple instantiation of the model for 3 classes of rectangles. Top: class-specific intensity distributions with means marked by solid lines. Middle: class-specific feature vectors. Bottom: 3 example data points, one for each class; corresponding intensities shown in top panel using dashed lines. (**C**) Differences between ‘1’ s and ‘0’ s in MNIST remain even after removing feature information by pixel shuffling. (**D**) We are presented with two new shuffled data points, overlaid on the class specific brightness distribution. Which class do the images belong to? Examples in C marked with colored dashed lines.

We start by defining a probabilistic generative model that describes how observations (stimuli) are generated (Fig. 2). According to our model, a stimulus **y** (e.g. the spectrogram of a specific utterance) with elements *y*_*d*_ (e.g. points in time-frequency space) belongs to a class *c* (e.g. the logatome ‘pap’). Stimuli are generated by multiplicatively combining class-specific features **W** with an intensity variable *z*. Class features (e.g., prototypical logatomes such as ‘pap’ and ‘zos’) are represented by the rows of matrix **W**. The intensity variable *z* is drawn from a *class*-*specific* distribution P(*z*|*c*), and Poisson noise is finally added to the observations (Fig. 2A). Here we chose to model shape using a prototypical class representation^8,15,18^ as it facilitates a fully probabilistic treatment of class and intensity information. Approaches that provide a decomposition of stimuli into different components^10,13,25^ pose additional analytical challenges, but may profit from the results provided here. The multiplicative effect of the intensity is motivated by previous work modeling contrast variations in images^6,7^. Since intensities must be positive, the Gamma distribution is a natural choice for P(*z*|*c*). Lastly, the Poisson variability is a canonical noise model for natural data, e.g. photon counts^26^ and neural spikes^27^. Its mathematical properties also facilitate a link to neural circuits^8,13,18^.

As a visual illustration of the generative model, Fig. 2B shows one simple artificial example in the visual domain. Stimuli belong to 3 classes which vary in their intensity distributions, with class 1 the dimmest and class 3 the brightest on average. Classes also vary in their shapes, modelled here as 2-D images of white boxes of varying sizes on a black background, normalized to sum to a constant. Individual observations are noisy versions of these prototypes, scaled by their intensity. Both the class identity and the intensity of any given stimulus are unknown and need to be inferred. Importantly, it is not only the location of the bright pixels that provides information about class identity; the overall brightness of the image, $\hat{y}$ = ∑_*d*_*y*_*d*_, is also informative about the class. Conversely, the intensity variable *z* depends not only on $\hat{y}$ but also on the total number of white pixels, which is class-specific. Hence knowing the class *c* helps to infer the value of *z* and vice versa.

Similar qualitative features are also present in real-world data. Figure 2C shows example images of written digits ‘0’ and ‘1’ from the MNIST dataset where the position of each pixel is shuffled to destroy shape information (i.e. features). The resulting images still look different across digits: the shuffled ‘0’ is brighter than the shuffled ‘1’. Hence, when presented with two other shuffled images one can make a decent guess about their classes despite missing spatial structure (Fig. 2D). This example suggests that intensity judgements may be generally useful, even when the assumptions of the model are not satisfied exactly.

### Inferring the class and intensity of stimuli under the generative model

Jointly inferring *c* and *z* given a stimulus **y** can be achieved by applying Bayes’s rule: ${\rm{P}}(c,z|{\bf{y}},\theta )\propto {\rm{P}}({\bf{y}}|c,z,\theta ){\rm{P}}(z|c,\theta ){\rm{P}}(c|\theta )$. *θ* is shorthand for parameters **W** and ***α***, ***β*** (the shape and rate parameters of the Gamma distributions, see Fig. 2A). While such a posterior usually requires approximate or numerical solutions, here it has a closed-form expression (see Supplementary Sec. S1):

1 $${\rm{P}}(c,z|{\bf{y}},\theta )=\frac{({\prod }_{d}{W}_{cd}^{{y}_{d}}){\rm{NB}}(\hat{y};{\alpha }_{c},\frac{1}{{\beta }_{c}+1})}{{\sum }_{c^{\prime} }({\prod }_{d}{W}_{c^{\prime} d}^{{y}_{d}}){\rm{NB}}(\hat{y};{\alpha }_{c^{\prime} },\frac{1}{{\beta }_{c^{\prime} }+1})}\cdot {\rm{Gam}}(z;{\alpha }_{c}+\hat{y},{\beta }_{c}+1),$$

where NB denotes the negative binomial distribution and $\hat{y}$ = ∑_*d*_*y*_*d*_.

Class judgements can be made by marginalizing over *z* the joint posterior above (see Supplementary Sec. S2):

2 $${\rm{P}}(c|{\bf{y}},\theta )=\frac{{\rm{N}}{\rm{B}}(\hat{y};{\alpha }_{c},\frac{1}{{\beta }_{c}+1})\exp ({\sum }_{d}{y}_{d}\,{\rm{l}}{\rm{n}}\,{W}_{cd})}{{\sum }_{c^{\prime} }{\rm{N}}{\rm{B}}(\hat{y};{\alpha }_{c^{\prime} },\frac{1}{{\beta }_{c^{\prime} }+1})\exp ({\sum }_{d^{\prime} }{y}_{d^{\prime} }\,{\rm{l}}{\rm{n}}\,{W}_{c^{\prime} d^{\prime} })}.$$

Despite its apparent complexity, this posterior is a straightforward generalization of the standard softmax function, which can be implemented neurally using well-understood winner-take-all (WTA) circuit dynamics^8,9,13,18,28^. It optimally combines information about the input shape, implicit in **y**, and its brightness $\hat{y}$. Moreover, if either cue is not instructive then the corresponding term cancels. If all classes have identical shape (i.e., **W** is the same across rows), then the posterior reduces to the negative binomial terms. Conversely, if all classes have the same intensity distribution, then we recover a traditional softmax consistent with previous work^8,11^. Similarly, intensity judgements are obtained by marginalizing the unknown class *c* (see Supplementary Sec. S2):

3 $${\langle z\rangle }_{{\rm{P}}(z|{\bf{y}},\theta )}=\sum _{c}{s}_{c}\frac{{\alpha }_{c}+\hat{y}}{{\beta }_{c}+1},$$

where *s*_*c*_ denotes the posterior probability of class *c* (Eq. 2). The expressions for marginal posteriors P(*c*|**y**, *θ*) and 〈*z*〉_P(*z*|**y**,*θ*)_ are relatively simple and local, suggesting that optimal inference in our model might be approximated by neural circuitry.

### Inferring the class and intensity of stimuli in a neural circuit

The form of the posterior for *c* (Eq. 2) is reminiscent of well-documented soft-WTA neural dynamics^8,9,13,18,28,29^. The similarity to neural circuits further increases in the limit when NB can be approximated by a Poisson distribution (see Supplementary Sec. S3):

$${\rm{P}}(c|{\bf{y}},\theta )\approx {s}_{c}=\frac{\exp \,({I}_{c})}{{\sum }_{c^{\prime} }\exp \,({I}_{c^{\prime} })};{I}_{c}=\sum _{d}{y}_{d}\,{\rm{l}}{\rm{n}}\,({W}_{cd}{\lambda }_{c})-{\lambda }_{c},$$

where *λ*_*c*_ = *α*_*c*_/*β*_*c*_.

**Figure 3:**
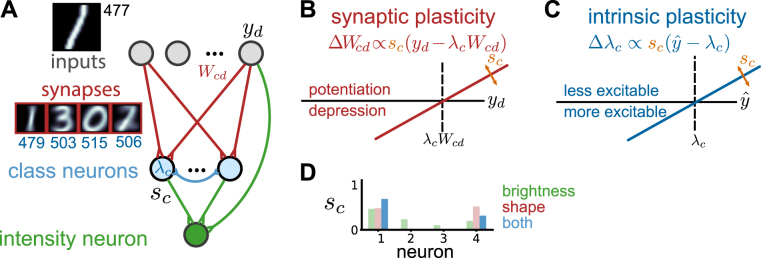
Neural circuit for inference and learning in face of class-specific intensity variations. (**A**) Circuit architecture: input neurons (gray) connect to the first processing layer (blue) via plastic synapses (red), with competition implemented by recurrent interactions. Neurons in this layer vary in their excitability *λ*, learned by IP (blue numbers). The activities *s*_*c*_ are combined across neurons and with the inputs to compute an estimate of the stimulus intensity. Insets show instantiation of the model after learning with 4 classes, trained on a subset of the MNIST dataset. (**B**) Optimal learning of model parameters via Hebbian and (**C**) IP (see text for details). (**D**) The outputs of the trained circuit for a novel ‘1’ input, either intact (in blue), or disrupted so as to preserve only shape (red) or brightness (green).

The inference of class label *c* can be approximated by a simple feedforward and neural circuit-like architecture (Fig. 3A). A layer of neurons coding for different class values *c* receive inputs **y** via synapses **W**_*c*_ and interact laterally and competitively to implement the softmax function. The excitability of these neurons is determined by an intrinsic parameter *λ*_*c*_ that reflects average brightness $\hat{y}$ of its preferred inputs. Jointly, the responses of the class neurons *s*_*c*_ encode not only the approximate Bayes-optimal guess for *c*, but also its associated uncertainty, in the form of the posterior probability^30^.

One potentially unrealistic aspect of the neural dynamics is the logarithmic nonlinearity affecting synaptic efficacies (but see^18^). As already shown by Keck *et al*.^8^, this issue can be eliminated by linearly approximating the logarithm. After this approximation, the expression for the input current to neuron *c* becomes ${I}_{c}=\sum _{d}{W}_{cd}{y}_{d}+\hat{y}\,\mathrm{log}\,{\lambda }_{c}-{\lambda }_{c}$ (see Supplementary Sec. S10). As before the primary effect of parameter *λ*_*c*_ is as a neuron-specific threshold. A more subtle effect involves a class-specific modulation of excitability that reflects the total input to the cell. Since the contribution of this term is typically small, we will focus primarily on the *λ*_*c*_ threshold regulation and its changes during learning.

The class posterior values *s*_*c*_ are combined linearly in a second layer to estimate the intensity of a stimulus *z* (Eq. 3), or quantities related to it such as the contrastive stress. We use expression ${\mathcal E} ={\langle z\rangle }_{{\rm{P}}(z|{\bf{y}},\theta )}-{\langle {\lambda }_{c}\rangle }_{{\rm{P}}(c|{\bf{y}},\theta )}$ as a sensible mathematical definition of contrastive stress: it starts from the original intuition of stress reflecting variations in intensity, and also accounts for the class-specific statistics of *z*. The posterior mean of the intensity *s*_*z*_ optimally combines direct stimulus information from the input layer with class judgements from the class layer to approximate ${\mathcal E}$ (see Fig. 3A and Supplementary Sec. S9):

4 $$ {\mathcal E} \approx {s}_{z}=K(\hat{y}-\sum _{c}{s}_{c}{\lambda }_{c}),$$

where *K* = 1/(*β* + 1) is a constant, and *β* approximates parameters *β*_*c*_, assumed to be similar across classes. While we do not necessarily think of contrastive stress estimation as a computation explicitly performed in the cortex, it is interesting to note that the final expression of the intensity estimation can still be performed using simple local operations.

The circuit only makes correct inferences when its synapses and intrinsic parameters reflect the true statistics of inputs. Computationally, these parameters could be learned from data by algorithms such as expectation maximization (EM)^31,32^. Exploiting the mathematical tractability of our model, we derived online parameter update rules that combine to approximate EM in a biologically-plausible way (see Supplementary Secs S4 and S5). The weight updates translate into a form of Hebbian synaptic plasticity, adapting the first layer synapses to reflect the shape-specific information for the corresponding class (Fig. 3B). The *λ*_*c*_ updates implement a form of intrinsic plasticity (IP) which adapts the excitability of the cell to reflect the average intensity of stimuli in that class (Fig. 3C):

5 $${\rm{\Delta }}{W}_{cd}={\varepsilon }_{W}{s}_{c}({y}_{d}-{\lambda }_{c}{W}_{cd});\,{\rm{\Delta }}{\lambda }_{c}={\varepsilon }_{\lambda }{s}_{c}(\hat{y}-{\lambda }_{c}),$$

where *ε*_*W*_ and *ε*_*λ*_ are small learning rates. The derived plasticity rules have the same fixed points as optimal EM learning (see Supplementary Sec. S4). They can be intuitively understood as trying to bring the values predicted by the generative model with parameters **W** and ***λ*** closer to the input values. In the case of Hebbian plasticity, the individual inputs *y*_*d*_ are compared with their expected values *λ*_*c*_*W*_*cd*_ (Fig. 3B). If the input is larger than expected then the synapse is potentiated, bringing the prediction closer to *y*_*d*_; if it is lower, synaptic depression occurs. The learning rule converges when the predictions are accurate (on average). Additionally, the magnitude of the synaptic changes is scaled by *s*_*c*_; the more likely it is that the stimulus belongs to the class, the larger the contribution of the current stimulus in updating the shape parameters. IP operates in a similar way, but for predictions about stimulus brightness, $\hat{y}$ (Fig. 3C): *λ*_*c*_ increases when the stimulus brightness is larger than expected and vice versa. The primary effect of this change on the neuron’s transfer function is as a threshold shift, which acts in a negative feedback loop, as in traditional phenomenological descriptions of homeostatic forms of IP. What is unique to our model is the fact that, similar to Hebbian plasticity, the magnitude of the excitability shift depends on the current activity of the neuron *s*_*c*_. Furthermore, the change in *λ*_*c*_ also induces a weaker positive feedback loop gain modulation of the total input to the neuron, as a non-homeostatic form of IP. Different experimental setups might preferentially reveal one or the other aspect of IP. Nonetheless, the fact that the change in excitability depends on the overall neural activation suggests possible routes for the experimental validation of the model (see Discussion).

One biologically implausible aspect of the above solution is that neural internal variables *λ*_*c*_ are needed when computing the activity in the second layer. While at first glance such non-locality seems to doom a neural implementation of optimal intensity judgements, the posterior can be approximated using a simple duplication of variables. The key idea is that a copy of *λ*_*c*_ is encoded in the synaptic connections *V*_*c*_ linking the first and second layers. These weights can be learned independently by Hebbian plasticity Δ*V*_*c*_ = *ε*_*V*_*s*_*c*_(*e* − *V*_*c*_), with learning rate *ε*_*V*_. Under the assumption that during learning the second layer is driven by feedforward inputs, i.e. ${s}_{z}=\hat{y}$, weights *V*_*c*_ will converge to *λ*_*c*_. Hence, it is possible to approximate optimal inference and learning for the Gamma-Poisson generative model with simple nonlinearities and local operations.

The resulting neural circuit self-organizes to optimally combine shape and brightness cues to make inferences. When trained using digits 0–3 in the MNIST dataset, the synapses learn to represent individual digits (Fig. 3A, red shaded images) while learned parameters *λ*_*c*_ reflect their average intensities (Fig. 3A, numbers in blue). When a new input is presented, e.g. a tilted ‘1’ digit with low brightness, the network correctly recognizes that it belongs to the class encoded by neuron 1 (Fig. 3D, blue bars). To highlight the fact that stimulus shape and brightness both matter for this judgement, we can disrupt the input so as to only preserve shape (Fig. 3D, red) or brightness (Fig. 3D, green) cues. Both manipulations negatively affect class identity judgements. Based on shape alone, the stimulus is equally likely to belong to the class represented by neurons 1 or 4. Hence it is the dimness of the stimulus that shifts the balance in favor of neuron 1 in the intact system. Similarly, brightness alone contains less information than shape; it cannot exclude any classes resulting in significantly higher class uncertainty.

### Inference and learning for visual data: classifying handwritten digits

**Figure 4:**
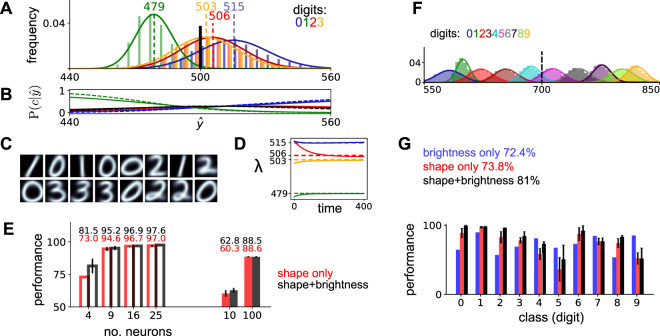
Brightness information improves handwritten digit classification. (**A**) Brightness histrograms for digits 0–3 from MNIST and corresponding gamma fits in solid lines; dashed vertical lines denote the mean brightness for each digit; vertical solid line marks the normalized brightness for the intensity-agnostic alternative neural circuit. (**B**) Posterior distribution of digit class using only brightness as a cue; solid lines are exact value, dashed are neural approximations (see Supplementary Sec. 3). (**C**) Learned weights **W** for *C* = 16 classes. (**D**) Solid lines show the evolution of estimated parameters (averaged across neurons tuned to the same digit); corresponding optimal values in dashed lines. Time measured as number of iterations through the training dataset. (**E**) Comparison of digit classification performance for circuits that ignore (red) or optimally take into account (black) class-specific variations in intensity, estimated using a Bayesian classifier using responses *s*_*c*_ as inputs. Chance performance is 25% for first 4 experiments (digits ‘0’–‘3’), and 10% for filled bars (digits ‘0’–‘9’). Error bars show s.d., estimated across 10 runs. (**F**) Variation of full handwritten digits in which brightness is artificially modulated in a class-specific way and (**G**) corresponding classification performance.

The proposed probabilistic model leverages class-specific brightness information to aid the classification of stimuli. Therefore it should outperform solutions that discard intensity information in a preprocessing step^8^. To test this hypothesis, we used digits 0–3 in MNIST and compared digit classification rates obtained using our circuit dynamics to those obtained by a circuit that requires normalized inputs (Fig. 4)^8^ (see Methods 1, Supplementary Sec. S7). Both models learn a representation of the (possibly normalized) data in an unsupervised manner, without access to class labels. To assess the quality of the emerging representations in terms of distinguishing different digits, we take the approach used in^8^ and train a Bayesian classifier with the class neuron responses *s*_*c*_ as input (see Supplementary Sec. S6). In this way, the classification performance jointly assesses the effects of inference and learning.

The MNIST dataset that we have chosen has limited variations in intensity across classes. Although the digit ‘1’ is well-separated in brightness space, digits ‘2’ and ‘3’ are virtually indistinguishable (Fig. 4A). These statistics are reflected in high confidence ‘1’ judgements when $\hat{y}$ is small, but higher uncertainty for intermediate and high values of $\hat{y}$ (Fig. 4B). Together with the fact that we are considering a relatively easy task (4 digit classes), we cannot expect the boost in performance to be large. Nonetheless, we do see significant performance benefits even in these conditions. Our model not only correctly learns prototypical digit shapes (Fig. 4C) and their corresponding average brightness values (Fig. 4D) but it also makes better digit class judgements (Fig. 4E).

The benefits of accounting for class-specific intensity differences are substantial when the number of classes is small (*C* = 4) but they decrease as the representation becomes more overcomplete (*C* = 25). Since classification performance is very high overall we might expect that limited improvements are due to reaching ceiling performance. To exclude this possibility, we repeated the experiment on the full MNIST dataset and found very similar results (see Supplementary Sec. 8). Our circuit outperforms a solution using input normalization in the complete scenario (solid bars in Fig. 4E, C = 10) but there are no differences when the representation is overcomplete (*C* = 100). This result suggests that, as the latent space increases, both circuits gain flexibility in encoding the statistics of the input, and brightness becomes less informative about class. It also suggests that our circuit is particularly efficient at capturing the true input statistics when neural resources are scarce.

We expect that explicitly taking into account class-specific intensity variations would prove particularly computationally advantageous when the correlation between class and brightness is high. To investigate the full potential of intensity-dependent inference and learning, we would need a dataset in which these dependencies are particularly strong. Unfortunately, traditional image datasets aim to minimize such differences by construction. To circumvent this issue, we decided to manipulate the intensity statistics of the standard MNIST dataset to artificially increase the dependence between brightness and stimulus class (by brightening the image foreground in a digit-specific way, see Fig. 4F and Methods 1). We further increase the task difficulty by considering all 10 digits. As before, the model learns accurate representations of the modified stimuli with only *C* = 20 classes (see Supplementary Sec. 8). The classification performance of individual digits is generally high, with the IP circuit generally outperforming classifiers based on single cues, either brightness or shape alone. The exception are the digits ‘4’, ‘7’ and ‘9’ where the network converges to inaccurate average brightness estimates. The poor learning for these classes is possibly due to their strong shape overlap, which could be resolved by increasing the number of class neurons. Nonetheless, after combining performance across all digits (Fig. 4G right) the intact circuit massively outperforms the alternatives (by 7%). These results confirm that, if the statistics of the inputs exhibit strong class-intensity dependencies, then it is computationally advantageous to explicitly account for them.

### Inference and learning for auditory data: estimating contrastive stress

**Figure 5:**
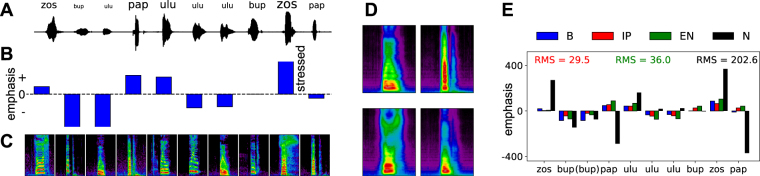
Estimating the contrastive stress of spoken logatomes and classification results. (**A**) Example data, which consists of a waveform sequence combining several logatomes, with different levels of stress, marked by the size of text above. (**B**) The Bayes-optimal stress estimate ${ {\mathcal E} }^{B}$ for the sentence in panel A. (**C**) Log-mel spectrograms of the waveforms in panel A, provided as inputs to the neural circuit. (**D**) Learned synaptic weights. (**E**) Four estimates of the stress of the test sentence in (**C**) ${ {\mathcal E} }^{B}$, ${ {\mathcal E} }^{IP}$, ${ {\mathcal E} }^{N}$, and ${ {\mathcal E} }^{EN}$. The colored text represents the root-mean-square distance of the latter three estimates from the Bayes-optimal ${ {\mathcal E} }^{B}$. X-axis shows the classification output for each of the logatomes in the sentence, with incorrect classifications in parentheses. These are typical results for this experiment.

While so far we have focused on class judgements, our inference procedure can also make intensity judgements for individual stimuli. While this computation may not be of interest for handwritten digits, it is of critical importance in the auditory domain, e.g. when estimating the contrastive stress of speech^1–4^. Hence, we use auditory data to investigate the utility of our model in making stress judgements. Figure 5A illustrates a particular version of this problem, based on the OLLO logatome database^5^ (see Methods 2). A speaker produces a sentence received by a listener. The speaker’s vocabulary comprises four logatome classes: ‘bup’, ‘pap’, ‘zos’, and ‘ulu’. The sentence includes 10 utterances of these logatomes, with varying levels of emphasis. The listener’s goal is to classify the logatomes in the sentence and to estimate their intensity, i.e. to estimate the contrastive stress of the sentence (Fig. 5B).

To accomplish that goal, we trained our network on inputs given as log-mel spectrograms of the individual logatomes (Fig. 5C, see Methods 2 for preprocessing details), reflecting patterns of neural activity as received by the primary auditory cortex^33^. The network had *C* = 4 class neurons. It was trained through a combination of synaptic plasticity and IP, as derived above. At the end of training, individual class neurons were each tuned to individual logatome classes, and weights **W**_*c*_ resembled general templates for each class of logatome (Fig. 5D; from the upper left box, going clockwise ‘ulu’, ‘pap’, ‘bup’, and ‘zos’). We then tested the network’s performance on contrastive stress estimation, as well as on classification, using a test sentence constructed from left-out data.

We compared the contrastive stress estimate of our model (Eq. 4, estimates marked ‘IP’/red in Fig. 5E), to several alternative estimators (see Supplementary Sec. S9). As lower bounds on performance we considered two *ad hoc* solutions including the naive estimate ${ {\mathcal E} }^{N}=\hat{y}-\sum _{n}{\hat{y}}^{(n)}/N$ discussed in the introduction (‘N’,/black), and an improved version, which approximates the expected intensity of a logatome as its brightness, i.e. ${\langle z\rangle }_{{\rm{P}}(z|{\bf{y}},\theta )}\approx \hat{y}$, and compares it to the average class intensity ${\langle {\lambda }_{c}\rangle }_{{\rm{P}}(c|{\bf{y}},\theta )}$ (‘EN’/green). This estimator has access to the ground-truth average brightnesses of individual logatome classes (i.e. all *λ*_*c*_), but deals sub-optimally with the map between intensity and brightness. Lastly, we used the exact optimal Bayesian estimate, ${ {\mathcal E} }^{B}$, for an upper bound on performance (‘B’/blue). We found that our model exhibits good performance on classification (the x-axis in Fig. 5E), despite the small size of the first layer. It correctly classified all logatomes except the third, where it mistook a weak ‘ulu’ for a ‘bup’. When estimating contrastive stress, the neural circuit implementation ${ {\mathcal E} }^{IP}$ is consistently close to the optimal Bayesian estimate ${ {\mathcal E} }^{B}$, being the least accurate only for the third logotome, which was misclassified. This suggests a relatively limited detrimental effect due to locality constraints and other neural approximations. As expected, the naive estimator ${ {\mathcal E} }^{N}$ differs wildly from ${ {\mathcal E} }^{B}$, particularly for the first, fourth, ninth, and tenth logatomes. The more sophisticated *ad hoc* estimator ${ {\mathcal E} }^{EN}$ performs closer to the optimum than ${ {\mathcal E} }^{N}$, but the neural implementation ${ {\mathcal E} }^{IP}$ was found to be the closest overall, as measured by the root-mean-square (RMS) distance between the stress estimate and the Bayes-optimal estimate ${ {\mathcal E} }^{B}$. This result reflects the fact that the brightness $\hat{y}$ and the expected intensity ${\langle z\rangle }_{{\rm{P}}(z|{\bf{y}},\theta )}$ are different quantities. While the estimates ${ {\mathcal E} }^{IP}$ and ${ {\mathcal E} }^{EN}$ are accurate approximations to the optimal estimate, the latter requires label information in order to calculate the average brightnesses *λ*_*c*_ of the dataset. The circuit also produces an accurate estimate, but learns the *λ*_*c*_ unsupervised (i.e. without labels).

## Discussion

Sensory perception needs to reconcile seemingly conflicting goals. On the one hand, salient features in the input should be identified regardless of stimulus intensity^34,35^. On the other hand, brightness information should be preserved for intensity judgements. While neural correlates of the first task have received considerable attention^7,12^, how neural circuits estimate stimulus intensity remains unclear. Here, we have argued that there are systematic dependencies between the class and intensity of sensory stimuli. We have derived a set of local inference and learning rules that can exploit class-intensity dependencies to approximate optimal judgements about such stimuli, and illustrated the benefits of the model on examples in the auditory and visual domains. We have further argued that this solution can be well approximated in a plausible neural circuit, where the interaction between specific forms of synaptic and intrinsic plasticity implement approximately optimal learning. It might be possible to derive other neural circuits for optimal learning of class and intensity inference. However, the interplay between synaptic plasticity and IP derived here (A) naturally emerges for such tasks, and (B) represents sensory statistics (shape and brightness) with sensible neural correlates (synaptic weights and neural excitabilities).

Although well-documented experimentally, the diversity of plasticity mechanisms co-active in cortical circuits remains a theoretical puzzle. This lack of functional understanding is particularly obvious when it comes to activity-dependent changes in neural excitability^36^, with the role of IP during learning still under debate^17,18,37–40^. In particular, the interaction between IP and synaptic plasticity has so far eluded analytical treatment. The traditional role of IP is viewed as a simple negative feedback loop that regulates average firing rates to keep overall activity close to a target value. Furthermore, recent work has suggested that sparsity-enforcing forms of IP may guide synaptic learning towards efficient neural representations^17^, and normative interpretations of (non-) homeostatic forms of IP have been proposed in the context of optimal memory recall^39^. Here we have expanded on these results by deriving the Bayes-optimal interaction between IP and synaptic plasticity as a key component of sensory learning.

Our IP rule differs from past proposals in that the target of regulation is average input currents rather than output firing rates. Furthermore, changes in excitability are both positive- and negative-feedback and gated by output activity. While different experimental manipulations reveal either homeostatic or non-homeostatic forms of IP^41^, directly validating these predictions is challenging. Independently manipulating the input and output statistics of the neuron is impossible in traditional IP experiments, which globally interfere with both inputs and outputs, typically by bath application of a drug^19,20^. Nonetheless, techniques that locally manipulate pre- and post-synaptic activity will open new avenues for testing our model experimentally. One very particular aspect of the model is that the excitability of neurons varies across the population, reflecting input statistics. In particular, neural excitability represents the mean intensity of the stimulus class that the neuron represents. This is unlike past IP models which assume that the target firing rate for IP is the same across neurons, set by energetic efficiency constraints^17,37,38^. Our model seems better aligned with biological findings, which suggest that neurons do not share a target firing rate but that cortical circuits have broad average firing rate distributions. Our approach could thus provide a computational account for systematic differences in excitability across neurons seen experimentally^42^.

From a computational perspective, our generative model provides a principled treatment of class-specific intensity variations, a ubiquitous feature of realistic datasets. With very limited computational overhead, the use of intensity as an additional cue improves the unsupervised learning of input statistics. The learned intensity statistics facilitate subsequent semi-supervised learning and classification, e.g. of MNIST digits or OLLO logatomes. Moreover, intensity itself can be estimated, and we illustrated one potential application of intensity estimation on acoustic data. The explicit use of syllable stress labels in modern automatic speech recognition (ASR) dictionaries may be taken as evidence for the importance of acoustic stress and intensity information in general^43,44^. For example, if a set of words are related but exhibit different stress patterns (e.g. ‘photograph’, ‘photographer’, ‘photographic’), then the consideration of syllable stress can improve recognition. However, ASR systems do not autonomously learn to infer stress estimates from data, whereas our approach specifies an unsupervised and statistically-grounded method to do so^45^].

## Methods

### M1. MNIST experiments

Figure 4 compares the classification performances on two different MNIST datasets which either use normalized inputs (Fig. 4A–E) or which incorporate class-specific brightness information (Fig. 4F,G). The difference between the inference procedures was that one required normalized inputs^8^ and classifies based on shape alone, while the other could learn class-specific brightnesses. Here we present the details for data preprocessing and the basic procedures used for training and testing, with corresponding pseudocode in Supplementary Sec. S7.

#### MNIST details: no class-dependent brightening

The shape-only inference requires inputs **y**^SA^ to be normalized to a constant *A*, with individual input elements greater than one^8^ (Fig. 4A–E):

6 $${y}_{d}^{{\rm{SA}}}=(A-D){\tilde{y}}_{d}^{{\rm{SA}}}/\sum _{d^{\prime} =1}^{D}{\tilde{y}}_{d^{\prime} }^{{\rm{SA}}}+1,$$

where *D* is the dimensionality of **y**^SA^, and ${\tilde{{\bf{y}}}}^{{\rm{SA}}}$ is the raw data; for the version of the dataset without class-dependent brightening (Fig. 4A–E) we use *D* = 400 (20 × 20 pixel images) and *A* = 500.

Our model receives input data **y**^IP^ that does not need to be normalized. To facilitate a fair comparison of the networks, we analogously preprocessed the MNIST dataset while preserving the class-specific brightness information in it. Specifically, we computed the brightness of raw MNIST data with respect to the average brightness of all data points: $f=\widehat{\tilde{y}}/{\langle \widehat{\tilde{y}}\rangle }_{{\rm{P}}(\widehat{\tilde{y}})}$. We then normalized each data point as we did for the shape-only circuit (Eq. 6, with *A* = 450), but amplified or dimmed the foreground of the image, depending on its original brightness, **y**^IP^ = (**y**^SA^ − 1) *f* + 1. The resulting dataset **y**^IP^ has an average brightness of 500 (see Fig. 4A) and all pixels are greater than 1. However, it preserves the brightness information from the raw MNIST dataset. We initialized the weights of both circuits ${{\bf{W}}}_{{\rm{IP}}}^{{\rm{init}}}$ and ${{\bf{W}}}_{{\rm{SA}}}^{{\rm{init}}}$ to randomly chosen preprocessed data points from the training set. Learning rates were *ε*_*W*_ = 1 × 10^−5^ and *ε*_*λ*_ = 1 × 10^−4^ for the version using unnormalized inputs and *ε* = 1 × 10^−3^ when learning based on shape alone^8^.

#### MNIST details: brightness-enhanced

We normalized **y**^SA^ as before (Eq. 6), but with *A* = 700. Our input to the IP network **y**^IP^, however, had its brightness enhanced depending on its class. We defined a vector *v*(*l*) that takes the label of a data point *l* as input *v*(*l*) = [2.3, 3.4, 3.3, 4.0, 4.8, 5.3, 5.9, 6.7, 6.9, 7.5]. We then normalized MNIST (Eq. 6, with *A* = 450) and amplified the image foregrounds depending on their original brightness and their class (i.e. their label), **y**^IP^ = (**y**^SA^ − 1) (*f* + *v*(*l*) + 1). The final dataset **y**^IP^ had an average brightness of 700 (see Fig. 4F) and all pixels greater than 1 (Fig. 4F,G).

We initialized $${{\bf{W}}}_{{\rm{IP}}}^{{\rm{init}}}$$ by taking the average of all data points and adding Poisson noise, $${{\bf{W}}}_{{\rm{IP}}}^{{\rm{init}}}=\sum _{n}{{\bf{y}}}^{(n)}/N+0.1X;$$$$X\sim {\rm{Poiss}}(X;1)$$, where *N* is the number of data points in the training set. Also, we initialized *λ*_*c*_ by drawing uniform random numbers between 550 and 850. These results indicate that the IP circuit can learn parameters of the dataset for a variety of initialization conditions.

For our proposed model we set the learning rates to *ε*_*W*_ = 1 × 10^−6^ and *ε*_*λ*_ = 1 × 10^−5^. The learning results remained qualitatively similar with changes in these values (see Fig. S5C,D), indicating that the model performance does not depend strongly on these parameters. One constraint is that one should make sure to prevent negative parameter values during training. For the shape-only circuit^8^, we set the learning rate to *ε* = 1 × 10^−3^, as before.

#### Parameter learning in the Gamma-Poisson model

Given a data point **y**^(*n*)^, we calculate the posterior over classes *s*_*c*_ and update the weights *W*_*cd*_ and intrinsic parameters *λ*_*c*_ by evaluating the relevant equations in the main text. We iteratively compute these quantities for every data point in the training set, and for some number of iterations over the training set. The behavior of these learning rules is qualitatively illustrated in Fig. 3B,C. Supplementary Sec. S7 presents formal pseudocode for this training process.

#### Testing the learned representation

The training of the models was done in an unsupervised fashion. However, to evaluate the quality of the learned representations we used the first layer outputs of the circuit **s**_1:*C*_ as inputs to a Bayesian classifier^8^ (see Supplementary Sec. S6). We ran these experiments in a semi-supervised setting where the representation was learned using inputs alone, and only a small fraction of the associated class labels (*L* = 30 examples for both MNIST and OLLO; for reference this is approximately 0.5% of the total number of labels in MNIST) were used to train the classifier. Performance was assessed using labeled test data.

### M2. OLLO experiment

The Oldenburg Logatome Corpus (OLLO)^5^ is a freely-available online database of acoustic data. It comprises logatomes that are nonsensical combinations of consonants (C) and vowels (V). There are 150 different combinations of CVCs and VCVs. Each is spoken by 50 different speakers: 25 men and 25 women; 40 from different regions of Germany and 10 from France. Each speaker produces each logatome in six different versions: ‘normal’, ‘fast’, ‘slow’, ‘loud’, ‘soft’, and ‘question’. The dataset we used for our experiments is a subset comprising logatomes ‘ulu’, ‘pap’, ‘zos’, ‘bup’, spoken in the ‘normal’ version, and only from the 40 German speakers. We used the Python package librosa (visit https://librosa.github.io/) to compute log-mel spectrograms of the audio files, using a sample rate of 16000 Hz, a hop length of 160, and setting the number of audio samples between successive onset measurements to 400. The spectrograms used 128 channels on the frequency axis.

Since our probabilistic model requires inputs **y**^(*n*)^ to have constant dimensionality, we trimmed spectrograms so that they had equivalent temporal durations. In particular, we used the 20 time bins containing the highest energy across all frequencies. We then calculated the time axis center of mass (COM) of those 20 columns and trimmed the spectrograms at 50 columns to either side of the COM. If we could not trim the spectrograms in this manner, i.e. if a logatome was pronounced very close to the beginning or end of a recording, then we discarded the data point. If the trimmed spectrograms contained less than 65% of the energy that was in the original spectrogram, then we also discarded the data point. Finally, we shifted and scaled the data **y**^(*n*)^ such that we can accurately approximate negative binomial distributions as Poisson (see Supplementary Sec. S3). This preprocessing procedure resulted in 310 valid log-mel spectrograms that we used for training (232 datapoints) and testing (78 datapoints). To construct the sentence shown in Fig. 5C, we chose ten random spectrograms from the testing set, with at least two examples of each logatome class. We initialized weights **W** by choosing four preprocessed data points and set the intensity parameters to their corresponding brightnesses. Learning rates were *ε*_*W*_ = 1 × 10^−6^ and *ε*_*λ*_ = 1 × 10^−2^.

## Electronic supplementary material

Supplementary Information
