# Supplementary material for: Optimal neural inference of stimulus intensities
Source: Sci Rep. 2018 Jul 3;8:10038. doi: 10.1038/s41598-018-28184-5 (PMC6030062; doi:10.1038/s41598-018-28184-5)
Supplement: Supplementary file 1 — Supplementary Information [file 41598_2018_28184_MOESM1_ESM.pdf]

Supplementary information: Optimal neural inference of stimulus intensities

Travis Monk[1,*], Cristina Savin[2], and Jörg Lücke[1]

[1]Cluster of Excellence Hearing4all, University of Oldenburg, Oldenburg 26129, Germany
[2]Center for Neural Science and Center for Data Science, NYU, New York 10003, USA
[*]travis.monk@uni-oldenburg.de

ABSTRACT

Supplementary Information S1: Full derivation of tractable joint class and intensity inference. S2: Derivation of closed-form marginal inference and compact expression of mean intensity. S3: Neural approximation of classification posterior. S4: Derivation showing neural plasticity rules have the same fixed points as maximum-likelihood EM learning. S5: Numerical verification of mathematical derivation in S4. S6: Overview of the Bayesian classifier that returns classification rate from learning results. S7: Pseudocode for training and testing the IP circuit on a dataset. S8: Full MNIST learning results on datasets with and without artificially-added class-specific brightness. S9: Formal definitions of contrastive stress. S10: Biologically plausible approximations for class inference and learning.

S1. Closed-form joint inference in the Gamma-Poisson model

The generative model (Fig. 2) is defined as:

$$\mathrm{P}(c|\theta)=\frac{1}{C};\qquad \mathrm{P}(z|c,\theta)=\mathrm{Gam}(z;\alpha_c,\beta_c);\qquad \mathrm{P}(\mathbf{y}|c,z,\theta)=\prod_{d=1}^{D}\mathrm{Poiss}(y_d;zW_{cd}),$$

where θ is shorthand for model parameters $\mathbf{W}$, α, and β, and C is the total number of classes. To avoid ambiguity in scales, and without loss of generality, we constrain the weights $\mathbf{W}_c$ to sum to a constant, $\sum_d W_{cd}=1$. Supplementary sec. S4 shows that our online Hebbian plasticity rule, at convergence, meets this constraint, as do empirical simulation results (see Fig. S3).

The joint posterior is obtained by applying Bayes' rule:

$$\mathrm{P}(c,z|\mathbf{y},\theta)=\frac{\mathrm{P}(\mathbf{y}|c,z,\theta)\mathrm{P}(z|c,\theta)\mathrm{P}(c|\theta)}{\sum_{c'}\int\mathrm{P}(\mathbf{y}|c',z,\theta)\mathrm{P}(z|c',\theta)\mathrm{P}(c'|\theta)dz}.$$

Assuming a flat prior for c, this expression becomes:

$$\mathrm{P}(c,z|\mathbf{y},\theta)=\frac{\mathrm{P}(\mathbf{y}|c,z,\theta)\mathrm{P}(z|c,\theta)}{\sum_{c'}\int\mathrm{P}(\mathbf{y}|c',z,\theta)\mathrm{P}(z|c',\theta)dz}.$$

Inserting the conditional probabilities $\mathrm{P}(\mathbf{y}|c,z,\theta)$ and $\mathrm{P}(z|c,\theta)$, the joint posterior becomes:

$$\mathrm{P}(c,z|\mathbf{y},\theta)=\frac{\left(\prod_d W_{cd}{}^{y_d}\right)z^{\hat{y}+\alpha_c-1}\exp(-z(\beta_c+1))\beta_c^{\alpha_c}\Gamma(\alpha_c)^{-1}}{\sum_{c'}\left(\prod_d W_{c'd}{}^{y_d}\right)\int z^{\hat{y}+\alpha_{c'}-1}\exp(-z(\beta_{c'}+1))\beta_{c'}^{\alpha_{c'}}\Gamma(\alpha_{c'})^{-1}dz}.$$

where $\hat{y}=\sum_d y_d$. Introducing the factors $(\beta_c+1)^{\hat{y}+\alpha_c}$ and $\Gamma(\hat{y}+\alpha_c)^{-1}$, we can recognize the integrand in the denominator as a Gamma distribution:

$$=\frac{\left(\prod_d W_{cd}{}^{y_d}\right)\frac{\beta_c^{\alpha_c}}{(\beta_c+1)^{\hat{y}+\alpha_c}}\frac{\Gamma(\hat{y}+\alpha_c)}{\Gamma(\alpha_c)}z^{\hat{y}+\alpha_c-1}\exp(-z(\beta_c+1))\frac{(\beta_c+1)^{\hat{y}+\alpha_c}}{\Gamma(\hat{y}+\alpha_c)}}{\sum_{c'}\left(\prod_d W_{c'd}{}^{y_d}\right)\frac{\beta_{c'}^{\alpha_{c'}}}{(\beta_{c'}+1)^{\hat{y}+\alpha_{c'}}}\frac{\Gamma(\hat{y}+\alpha_{c'})}{\Gamma(\alpha_{c'})}\int z^{\hat{y}+\alpha_{c'}-1}\exp(-z(\beta_{c'}+1))\frac{(\beta_{c'}+1)^{\hat{y}+\alpha_{c'}}}{\Gamma(\hat{y}+\alpha_{c'})}dz},$$

which must integrate to 1. The corresponding term in the numerator is also a Gamma distribution, so the joint posterior becomes:

$$\mathrm{P}(c,z|\mathbf{y},\theta)=\frac{\left(\prod_d W_{cd}{}^{y_d}\right)\frac{\beta_c^{\alpha_c}}{(\beta_c+1)^{\hat{y}+\alpha_c}}\frac{\Gamma(\hat{y}+\alpha_c)}{\Gamma(\alpha_c)}}{\sum_{c'}\left(\prod_d W_{c'd}{}^{y_d}\right)\frac{\beta_{c'}^{\alpha_{c'}}}{(\beta_{c'}+1)^{\hat{y}+\alpha_{c'}}}\frac{\Gamma(\hat{y}+\alpha_{c'})}{\Gamma(\alpha_{c'})}}\mathrm{Gam}(z;\alpha_c+\hat{y},\beta_c+1).$$

After multiplying the numerator and denominator by $(\hat{y}!)^{-1}$, we recognize the ratios in the numerator and denominator as negative binomial (NB) distributions, which yields the final expression for the joint posterior:

$$\mathrm{P}(c,z|\mathbf{y},\theta) = \frac{\left(\prod_d W_{cd}{}^{y_d}\right)\mathrm{NB}\left(\hat{y};\alpha_c,\frac{1}{\beta_c+1}\right)}{\sum_{c'}\left(\prod_d W_{c'd}{}^{y_d}\right)\mathrm{NB}\left(\hat{y};\alpha_{c'},\frac{1}{\beta_{c'}+1}\right)}\mathrm{Gam}(z;\alpha_c+\hat{y},\beta_c+1). \tag{S1}$$

S2. Closed-form marginal inference

We obtain the marginal posterior over classes $\mathrm{P}(c|\mathbf{y},\theta)$ by integrating the joint posterior Eq. S1 over z:

$$\mathrm{P}(c|\mathbf{y},\theta) = \frac{\mathrm{NB}\left(\hat{y};\alpha_c,\frac{1}{\beta_c+1}\right)\exp\left(\sum_d y_d \ln W_{cd}\right)}{\sum_{c'}\mathrm{NB}\left(\hat{y};\alpha_{c'},\frac{1}{\beta_{c'}+1}\right)\exp\left(\sum_d y_d \ln W_{c'd}\right)}, \tag{S2}$$

where we have rewritten $\prod_d W_{cd}{}^{y_d}$ as $\exp\left(\sum_d y_d \ln W_{cd}\right)$. Similarly, the posterior for the intensity z is:

$$\mathrm{P}(z|\mathbf{y},\theta) = \sum_c \mathrm{P}(z|c,\mathbf{y},\theta)\mathrm{P}(c|\mathbf{y},\theta).$$

This expression is computed using the previously-derived expression for $\mathrm{P}(c|\mathbf{y},\theta)$ (Eq. S2). We find the remaining factor $\mathrm{P}(z|c,\mathbf{y},\theta)$ by comparing terms with Eq. S1:

$$\mathrm{P}(z|c,\mathbf{y},\theta) = \mathrm{Gam}(z;\alpha_c+\hat{y},\beta_c+1). \tag{S3}$$

The intensity posterior mean also has a simple form:

$$\langle z\rangle_{\mathrm{P}(z|\mathbf{y},\theta)} = \sum_c \int z\,\mathrm{P}(z|c,\mathbf{y},\theta)\mathrm{P}(c|\mathbf{y},\theta)dz.$$

Inserting $s_c = \mathrm{P}(c|\mathbf{y},\theta)$, Eq. S3, and rearranging yields:

$$\langle z\rangle_{\mathrm{P}(z|\mathbf{y},\theta)} = \sum_c s_c \int z\,\mathrm{Gam}(z;\alpha_c+\hat{y},\beta_c+1)dz.$$

Identifying the integral as the mean of a Gamma distribution we obtain:

$$\langle z\rangle_{\mathrm{P}(z|\mathbf{y},\theta)} = \sum_c s_c \frac{\alpha_c+\hat{y}}{\beta_c+1}.$$

Notice that the expected intensity, given a stimulus is not equivalent to the stimulus brightness $\hat{y}$; the intensity is a hidden variable to be inferred, whereas the brightness is an observable quantity. However, the expected intensity, *given the class* is equivalent to the expected brightness of that class:

$$\langle z\rangle_{\mathrm{P}(z|c,\theta)} = \left\langle \langle z\rangle_{\mathrm{P}(z|\hat{y},c,\theta)}\right\rangle_{\mathrm{P}(\hat{y}|c,\theta)} = \langle(\alpha_c+\hat{y})/(\beta_c+1)\rangle_{\mathrm{P}(\hat{y}|c,\theta)} = \frac{\alpha_c}{\beta_c+1} + \frac{1}{\beta_c+1}\langle\hat{y}\rangle_{\mathrm{P}(\hat{y}|c,\theta)}.$$

Identifying $\langle z\rangle_{\mathrm{P}(z|c,\theta)} = \alpha_c/\beta_c \equiv \lambda_c$ and solving for $\langle\hat{y}\rangle_{\mathrm{P}(\hat{y}|c,\theta)}$ yields:

$$\langle\hat{y}\rangle_{\mathrm{P}(\hat{y}|c,\theta)} = \alpha_c/\beta_c = \lambda_c = \langle z\rangle_{\mathrm{P}(z|c,\theta)}. \tag{S4}$$

S3. Softmax approximation to class inference

Eq. S2 can be simplified by approximating the negative binomial distribution as Poisson:

$$\lim_{\alpha_c\to\infty,\lambda_c=\text{const.}} \mathrm{NB}(\hat{y};\alpha_c,1/(\beta_c+1)) = \mathrm{Poiss}(\hat{y};\lambda_c),$$

where $\lambda_c \equiv \alpha_c/\beta_c$. In this limit, Eq. S2 becomes:

$$\mathrm{P}(c|\mathbf{y},\theta) \approx \frac{\exp(\sum_d y_d \ln(W_{cd}\lambda_c)-\lambda_c)}{\sum_{c'}\exp(\sum_{d'} y_{d'}\ln(W_{c'd'}\lambda_{c'})-\lambda_{c'})}, \tag{S5}$$

which is a softmax function with input $\sum_{d'} y_{d'}\ln(W_{cd'}\lambda_c)-\lambda_c$.

The approximation is accurate when $\alpha_c \gg \beta_c$, which does not necessarily hold for any given dataset. One way to verify the accuracy of the approximation S5 is to compare the exact and approximate class posteriors given only the brightness of the input (i.e. given no shape information). Canceling the W_{cd} terms from Eq. S2 yields:

$$\mathrm{P}(c|\hat{y},\theta) = \frac{\mathrm{NB}(\hat{y};\alpha_c,\frac{1}{\beta_c+1})}{\sum_{c'}\mathrm{NB}(\hat{y};\alpha_{c'},\frac{1}{\beta_{c'}+1})} \approx \frac{\mathrm{Poiss}(\hat{y};\lambda_c)}{\sum_{c'}\mathrm{Poiss}(\hat{y};\lambda_{c'})}.$$

The solid (exact) and dashed (approximation) lines in Fig. 4B (main text) and Fig. S5B (see Supplementary sec. 8) verify that the neural implementation is reasonably accurate for our data.

S4. Optimal and biological parameter learning

EM learning

Given a set of N data points $\mathbf{y}^{(n)}$, we seek the parameters $\theta = \{\mathbf{W},\lambda\}$ that maximize the data likelihood under our generative model. We use the expectation maximization (EM) formulation introduced in Dempster *et al* (1977) and Neal and Hinton (1998), and optimize the free-energy given by:

$$\mathscr{F}(\theta_{\mathrm{t}},\theta_{\mathrm{t\text{-}1}}) = \sum_n\sum_{c'}\mathrm{P}(c'|\mathbf{y}^{(n)},\theta_{\mathrm{t\text{-}1}})(\ln\mathrm{P}(\mathbf{y}^{(n)}|c',\theta_{\mathrm{t}}) + \ln\mathrm{P}(c'|\theta_{\mathrm{t}})) + H(\theta_{\mathrm{t\text{-}1}}),$$

where $H(\theta_{\mathrm{t\text{-}1}})$ is the Shannon entropy of the posterior as a function of the previous parameter values.

The M-step update for parameters λ_c and W_{cd} is obtained by taking the partial derivative of $\mathscr{F}(\theta_{\mathrm{t}},\theta_{\mathrm{t\text{-}1}})$ w.r.t. each parameter and setting it to zero. The update rule for the intensity parameter $\lambda_{c,\mathrm{t}}$ becomes:

$$\frac{\partial\mathscr{F}(\theta_{\mathrm{t}},\theta_{\mathrm{t\text{-}1}})}{\partial\lambda_{c,\mathrm{t}}} = 0 \quad \Rightarrow \quad \lambda_{c,\mathrm{t}} = \frac{\sum_n\mathrm{P}(c|\mathbf{y}^{(n)},\theta_{\mathrm{t\text{-}1}})\hat{y}^{(n)}}{\sum_n\mathrm{P}(c|\mathbf{y}^{(n)},\theta_{\mathrm{t\text{-}1}})}. \tag{S6}$$

The corresponding update for the $\mathbf{W}$ parameters is obtained in a similar manner, with the additional constraint that $\sum_d W_{cd} = 1$ for all c, enforced using Lagrange multipliers Λ_c:

$$\frac{\partial\mathscr{F}(\theta_{\mathrm{t}},\theta_{\mathrm{t\text{-}1}})}{\partial W_{cd,\mathrm{t}}} + \frac{\partial}{\partial W_{cd,\mathrm{t}}}\sum_{c'}\Lambda_{c'}\left(\sum_{d'}W_{c'd',\mathrm{t}} - 1\right) = 0 \quad \Rightarrow \quad W_{cd,\mathrm{t}} = \frac{\sum_n y_d\mathrm{P}(c|\mathbf{y}^{(n)},\theta_{\mathrm{t\text{-}1}})}{\sum_d\sum_n y_d\mathrm{P}(c|\mathbf{y}^{(n)},\theta_{\mathrm{t\text{-}1}})}. \tag{S7}$$

Fig. S1 (see Supplementary sec. 5) presents numerical verification of Eqs. S6 and S7.

Online learning

The derivation of the synaptic learning rule is a straightforward generalization of the learning rule in Keck *et al* (2012). The weights of the neural circuit W_{cd} change according to:

$$\Delta W_{cd} = \varepsilon_W(s_c y_d - s_c\bar{W}_c\lambda_c W_{cd}), \tag{S8}$$

where ε_W is a small and positive learning rate, and $\bar{W}_c = \sum_d W_{cd}$. Keck *et al* (2012) showed that, if $\bar{W}_c$ is normalized at convergence, then the weights have the same fixed points as optimal EM learning. The same results apply here, as we now show that $\bar{W}_c$ converges to 1 (Monk *et al* (2016)).

Summing both sides of Eq. S8 over d:

$$\Delta\bar{W}_c = \varepsilon_W(s_c\hat{y} - s_c\lambda_c\bar{W}_c^2).$$

Assume that the weights have converged, and let the network observe a batch of N data points. The average change in $\bar{W}_c$ given the batch of N data points is:

$$\Delta\bar{W}_c^{(N)} = \frac{1}{N}\sum_n\varepsilon_W(s_c^{(n)}\hat{y}^{(n)} - s_c^{(n)}\lambda_c\bar{W}_c^2).$$

Assuming that the inputs $\mathbf{y}^{(n)}$ are drawn from a stationary distribution $\mathrm{P}(\mathbf{y}^{(n)})$, and assuming a small learning rate and a large batch size, we can accurately approximate the sum with an expectation:

$$\Delta\bar{W}_c^{(N)} \approx \varepsilon_W\left(\langle s_c\hat{y}\rangle_{\mathrm{P}(\mathbf{y}^{(n)})} - \lambda_c\bar{W}_c^2\langle s_c\rangle_{\mathrm{P}(\mathbf{y}^{(n)})}\right). \tag{S9}$$

Next we approximate the two expectations in Eq. S9. Inserting $s_c = \mathrm{P}(c|\mathbf{y}^{(n)}, \theta)$, the left expectation may be written as:

$$\langle s_c \hat{y} \rangle_{\mathrm{P}(\mathbf{y}^{(n)})} = \sum_{\mathbf{y}^{(n)}} \hat{y} \frac{\mathrm{P}(c, \mathbf{y}^{(n)}|\theta)}{\mathrm{P}(\mathbf{y}^{(n)}|\theta)} \mathrm{P}(\mathbf{y}^{(n)}).$$

If the true data distribution is the same as the distribution learned by the model, then $\mathrm{P}(\mathbf{y}^{(n)}|\theta)$ and $\mathrm{P}(\mathbf{y}^{(n)})$ cancel:

$$\langle s_c \hat{y} \rangle_{\mathrm{P}(\mathbf{y}^{(n)})} = \mathrm{P}(c|\theta) \sum_{\mathbf{y}^{(n)}} \hat{y} \mathrm{P}(\mathbf{y}^{(n)}|c, \theta). \tag{S10}$$

We can rewrite the sum as a conditional expectation:

$$\sum_{\mathbf{y}^{(n)}} \hat{y} \mathrm{P}(\mathbf{y}^{(n)}|c, \theta) = \sum_{\hat{y}} \hat{y} \sum_{\sum_d \mathbf{y}^{(n)} = \hat{y}} \mathrm{P}(\mathbf{y}^{(n)}|c, \theta) = \sum_{\hat{y}} \hat{y} \mathrm{P}(\hat{y}|c, \theta) = \langle \hat{y} \rangle_{\mathrm{P}(\hat{y}|c,\theta)} .$$

Using the tower property of conditional expectations and evaluating them for our generative model:

$$\langle \hat{y} \rangle_{\mathrm{P}(\hat{y}|c,\theta)} = \left\langle \langle \hat{y} \rangle_{\mathrm{P}(\hat{y}|z,c,\theta)} \right\rangle_{\mathrm{P}(z|c,\theta)} = \langle z \bar{W}_c \rangle_{\mathrm{P}(z|c,\theta)} = \bar{W}_c \lambda_c.$$

Inserting $\bar{W}_c \lambda_c$ for the sum in Eq. S10:

$$\langle s_c \hat{y} \rangle_{\mathrm{P}(\mathbf{y}^{(n)})} \approx \mathrm{P}(c|\theta) \bar{W}_c \lambda_c.$$

The right expectation in Eq. S9 is:

$$\langle s_c \rangle_{\mathrm{P}(\mathbf{y}^{(n)})} = \sum_{\mathbf{y}^{(n)}} \frac{\mathrm{P}(\mathbf{y}^{(n)}|c, \theta) \mathrm{P}(c|\theta)}{\mathrm{P}(\mathbf{y}^{(n)}|\theta)} \mathrm{P}(\mathbf{y}^{(n)}).$$

If the true data distribution is the same as the distribution learned by the model, then $\mathrm{P}(\mathbf{y}^{(n)}|\theta)$ and $\mathrm{P}(\mathbf{y}^{(n)})$ cancel:

$$\langle s_c \rangle_{\mathrm{P}(\mathbf{y}^{(n)})} = \mathrm{P}(c|\theta) \sum_{\mathbf{y}^{(n)}} \mathrm{P}(\mathbf{y}^{(n)}|c, \theta) = \mathrm{P}(c|\theta).$$

Inserting our expressions for $\langle s_c \hat{y} \rangle_{\mathrm{P}(\mathbf{y}^{(n)})}$ and $\langle s_c \rangle_{\mathrm{P}(\mathbf{y}^{(n)})}$ into Eq. S9:

$$\Delta \bar{W}_c^{(N)} \approx \varepsilon_W \mathrm{P}(c|\theta) \lambda_c \bar{W}_c \left(1 - \bar{W}_c\right).$$

This expression has stationary points at $\bar{W}_c = 1$ (stable) and 0 (unstable). Hence if the weights are initialized to be positive and the learning rate is sufficiently small, then $\bar{W}_c$ converges to the desired norm.

If we assume that the average weights $\bar{W}_c$ have converged to 1, then we can use the results of Keck *et al* (2012), who showed that the weights W_{cd} at convergence are approximately:

$$W_{cd}^{\mathrm{conv}} \approx \frac{\sum_n y_d^{(n)} s_c}{\sum_{d'} \sum_n y_{d'}^{(n)} s_c}. \tag{S11}$$

The update rules for λ_c take a similar form:

$$\Delta \lambda_c = \varepsilon_\lambda s_c (\hat{y} - \lambda_c), \tag{S12}$$

where ε_λ is another small positive learning rate. Consider the change in λ_c given a batch of N data points. Assuming that the inputs are drawn from a stationary distribution, and assuming a small learning rate and large batch size, we can approximate $\Delta \lambda_c^{(N)}$ with expectations:

$$\Delta \lambda_c^{(N)} \approx \varepsilon_\lambda \left(\langle s_c \hat{y} \rangle_{\mathrm{P}(\hat{y})} - \lambda_c \langle s_c \rangle_{\mathrm{P}(\hat{y})} \right).$$

The parameter dynamics have a stable fixed point at:

$$\lambda_c = \frac{\langle s_c \hat{y} \rangle_{\mathrm{P}(\hat{y})}}{\langle s_c \rangle_{\mathrm{P}(\hat{y})}}. \tag{S13}$$

Comparing the convergence expressions (Eqs. S11 and S13) with the EM updates (Eqs. S6 and S7), and inserting the definition $s_c = \mathrm{P}(c|\mathbf{y}, \theta)$, we see that the neural dynamics given in Eqs. S8 and S12 have the same fixed points as optimal EM learning. Fig. S2 (see Supplementary section 5) presents numerical verification of this result.

S5. Numerical verification of optimal and biological parameter learning

EM learning

As numerical verification of our EM derivations (see Supplementary sec. S4), Fig. S1 illustrates the evolution of parameters λ_c and W_{cd} yielded by the EM algorithm on artificial data. Our artificial data set consists of four classes of rectangles on a grid of 10×10 pixels. Rectangles from different classes have different sizes and positions and are represented by a generative vector W_c^{gen}.

We generate a data set by drawing a number $N = 2000$ of observations of W_c^{gen}, with each class equiprobable. We then draw a random variable z from a Gamma distribution with parameters α_c and β_c that depend on the class of each observation. Then, given W_c^{gen} and z, we create a data vector $\mathbf{y}^{(n)}$ by adding Poisson noise to each pixel. With a set of N data vectors $\mathbf{y}^{(n)}$, we then perform EM to find the parameters W_{cd} and λ_c that maximize the likelihood of the data set (at least locally). The E-step evaluates Eq. 2 (see main text) for each data vector, and the M-step evaluates Eqs. S6 and S7. Figure S1 shows that, after about five iterations, the EM algorithm has already accurately approximated the values of W_{cd} and λ_c that were used to generate the data set.

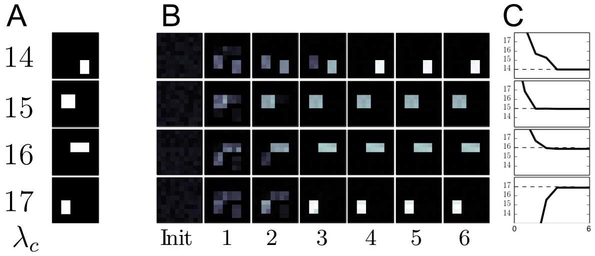

Figure S1. The EM algorithm learns the parameter values that were used to generate our artificial dataset. A: Four classes of rectangles represented by the vector W_c^{gen}, with the values of λ_c for each class displayed to the left. B: Evolution of the parameters W_{cd} for successive iterations of the EM algorithm. C: Evolution of the parameters λ_c, with dashed lines indicating the values from the data generating model. For these plots, we generated a data set of 2000 inputs. $W_c^{\text{gen}} = 100$ for white pixels and 1 for black pixels. The shape and rate parameters of the Gamma distributions, from the top class to the bottom, are $\alpha = [98, 112, 128, 144]$ and $\beta = [7, 7.5, 8, 8.5]$, giving $\lambda_c = \alpha_c/\beta_c = [14, 15, 16, 17]$.

Neural circuit learning

To verify our analytical results for neural circuit learning (see Supplementary sec. 4), we first investigated its learning using artificial data generated according to our generative model (the same dataset as used for Fig. S1). Fig. S2 displays the evolution of parameters λ_c and W_{cd} of the circuit. The circuit learns the parameters that were used to generate the data set. We also verified the learning results of the circuit on natural data using a subset of the MNIST dataset (the digits 0 through 3).

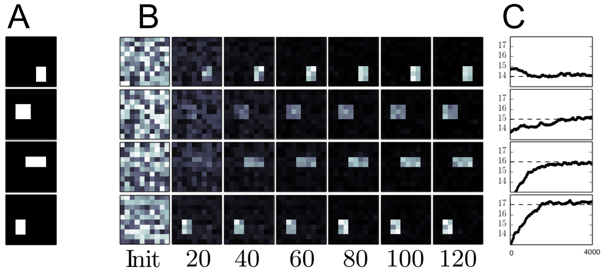

Figure S2. Using the same dataset from Fig. S1, the neural circuit also learns the parameter values that generated the dataset. A: Four classes of rectangles with the same values of λ_c as in Fig. S1. B: Evolution of the synaptic weights W_{cd} that feed each class neuron after 0, 20, 40, ..., 120 time steps, respectively. C: Evolution of the intrinsic parameters λ_c over 4000 time steps, with dashed lines indicating the values from the generative model. The neural circuit returns the values of W_{cd} and λ_c that were used to generate the data set, i.e. the parameter values that maximize the data likelihood. For this plot, $\varepsilon_W = \varepsilon_\lambda = .005$, $D = 100$ (for a 10x10 pixel grid), $C = 4$.

Fig. S3 presents typical learning results if a circuit with $C = 9$ class neurons is applied to this data. The circuit learns different writing styles for each digit, and the sum of the weights feeding each class neuron remained near 1 throughout training.

In further controls using this data, with weights explicitly normalized to 1, we did not observe significant differences compared to the unconstrained version. In all other numerical experiments we therefore used weights explicitly constrained to one because of practical advantages, such as simplified evaluation of the data log-likelihood throughout training (e.g., Fig. S4 D).

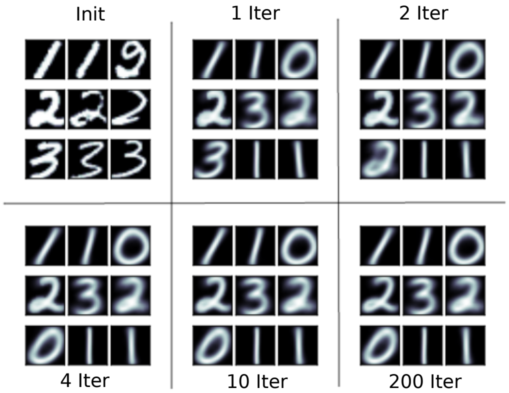

Figure S3. The evolution of the synaptic weights on a subset of MNIST. Weights feeding each of the $C = 9$ class neurons are shown after the stated number of iterations over the training set. We initialized the synaptic weights as randomly-chosen data points from the MNIST subset, normalized to 1 (upper-left). We confirmed that the sum of the weights feeding each class neuron remained close to 1 during learning.

S6. Bayesian classifier

We briefly review the Bayesian classifier from Keck *et al* (2012), which we used to assess the quality of learned neural representations of different stimuli. The general setup is that of hierarchical inference, in which the stimuli belong to a set of K given label classes, and are represented at the intermediate level as the activity of C class neurons (with neural dynamics as described above). For instance, for MNIST the class labels are individual digits '0'-'9' ($K = 10$) while individual cells ($C > K$) may specialize to encode different ways of writing the same digit.

Given the available labeled training data, we can estimate the likelihood, B_{ck}, of the class label being k, given the activation of neuron c as (see Keck *et al* (2012) for derivations):

$$B_{ck} = \frac{1}{L} \sum_{l=1}^{L} \mathrm{P}(c|\mathbf{y}^{(l)}, \theta),$$

where $\mathbf{y}^{(l)}$ denotes the L stimuli with label k.

Finally, the posterior over class labels can be approximated by applying Bayes' rule, and marginalising out c:

$$\mathrm{P}(k|\mathbf{y}^{(n)}, \theta) \approx \frac{\sum_c B_{ck} \mathrm{P}(\mathbf{y}^{(n)}|c, \theta)}{\sum_{k'} \sum_{c'} B_{c'k'} \mathrm{P}(\mathbf{y}^{(n)}|c', \theta)},$$

where, when $\bar{W}_c = 1$, becomes:

$$\begin{aligned}
\mathrm{P}(\mathbf{y}^{(n)}|c, \theta) &= \int \mathrm{P}(\mathbf{y}^{(n)}|c, z, \theta) \mathrm{P}(z|c, \theta) dz \\
&= \left(\prod_d \frac{W_{cd}^{y_d^{(n)}}}{y_d^{(n)}!} \right) \hat{y}^{(n)}! \mathrm{NB}(\hat{y^{(n)}}; \alpha_c, 1/(\beta_c + 1)).
\end{aligned}$$

Approximating the negative binomial as Poisson, we obtain:

$$\mathrm{P}(\mathbf{y}^{(n)}|c, \theta) \approx \left(\prod_d \frac{W_{cd}^{y_d^{(n)}}}{y_d^{(n)}!} \right) \lambda_c^{\hat{y}^{(n)}} \exp(-\lambda_c).$$

S7. Pseudocode for training and testing the IP circuit

Pseudocode describing the parameter learning procedure is shown in Alg. 1. Alg. 2 presents pseudocode for evaluating the classification rate of the IP circuit. We further used the Bayesian classifier in our visualization of the learned intrinsic parameters λ_c in Fig. 4 D. The label assignments for each learned class were assigned as MAP estimates from the Bayesian classifier. Each colored trace in Fig. 4 D corresponds to the average of the intrinsic parameters over all class neurons that correspond to a given digit.

Algorithm 1 Online learning training procedure

function SOFTMAX($\mathbf{y}_{\text{IP}}^{(n)}$, λ, W)
  $I_c \leftarrow -\lambda_c + \sum_d \mathbf{y}_{\text{IP}}^{(n)} \log(\lambda_c W_{cd})$
  $s_c \leftarrow \exp(I_c)/\sum_c \exp(I_c)$
  return s_c
function UPDATE WEIGHTS(s_c,$\mathbf{y}_{\text{IP}}^{(n)}$,$\lambda$,$W$)
  $W_{cd} \leftarrow \varepsilon_W s_c (y_{\text{IP},d} - \lambda_c W_{cd}) + W_{cd}$
  return W_{cd}
function UPDATE INTRINSICS(s_c,$\mathbf{y}_{\text{IP}}^{(n)}$,$\lambda$)
  $\lambda_c \leftarrow \varepsilon_\lambda s_c (\hat{y}_{\text{IP}} - \lambda_c) + \lambda_c$
  return λ_c
function NORMALIZE WEIGHTS(W)
  $W_{cd} \leftarrow W_{cd}/\sum_d W_{cd}$
  return W_{cd}
Initialize parameters
▷ Train circuit
for training set iterations **do**
  for training data points **do**
    $s_c = \text{SOFTMAX}(\mathbf{y}_{\text{IP}}^{(n)}, \lambda, W)$
    $W_{cd} = \text{UPDATE WEIGHTS}(s_c, \mathbf{y}_{\text{IP}}^{(n)}, \lambda, W)$
    $\lambda_c = \text{UPDATE INTRINSICS}(s_c, \mathbf{y}_{\text{IP}}^{(n)}, \lambda)$
    $W_{cd} = \text{NORMALIZE WEIGHTS}(W)$

Algorithm 2 Quantifying the quality of the learned representation

function BAYESIAN CLASSIFIER($y_{\text{IP}}^{(l)}$,L,λ,W)
  for labeled data points **do**
    $s_c^{(l)} = \text{SOFTMAX}(\mathbf{y}_{\text{IP}}^{(l)}, \lambda, W)$
  $B_{ck} \leftarrow \frac{1}{L} \sum_{l=1}^{L} s_c^{(l)}$
  return B
function CLASSIFY($\mathbf{y}_{\text{IP}}$,B,λ,W)
  $\text{P}(\mathbf{y}_{\text{IP}}|c,\lambda,W) \leftarrow \left(\prod_d W_{cd}^{y_d}\right) \lambda_c^{\hat{y}} \exp(-\lambda_c)/\Gamma(\hat{y}+1)$
  $\text{P}(k|\mathbf{y}_{\text{IP}},\lambda,W) \leftarrow \frac{\sum_c B_{ck} \text{P}(\mathbf{y}_{\text{IP}}|c,\lambda,W)}{\sum_{k'} \sum_{c'} B_{c'k'} \text{P}(\mathbf{y}_{\text{IP}}|c',\lambda,W)}$
  classification = $\max(\text{P}(k|\mathbf{y}_{\text{IP}},\lambda,W))$
  return classification
For every class k choose L training data points
$B = \text{BAYESIAN CLASSIFIER}(\mathbf{y}_{\text{IP}}^{(l)}, L, \lambda, W)$
for testing data points **do**
  classification = $\text{CLASSIFY}(\mathbf{y}_{\text{IP}}^{(n)}, B, \lambda, W)$
rate = $\sum_n$ (classification == labels) / N

S8. Full MNIST learning results

Standard MNIST

Fig. S4 presents typical learning results of the IP circuit with $C = 100$ class neurons applied to the standard full MNIST dataset. At convergence (after 400 training iterations) synaptic weights resemble different writing styles of individual digits (Fig. S4 B). The corresponding evolution of λ parameters is shown in Fig. S4 C. Almost all average intensity parameters are very close (within three intensity units) to their ground-truth values. The corresponding log-likelihood values under our generative model increase systematically during training (Fig. S4 D). The classification rate of the IP circuit is compared with that of the shape-alone circuit in Fig. 4 E (see main text). Overall, these results show that our approximate online learning procedure correctly captures the shape and average brightness of MNIST digit classes.

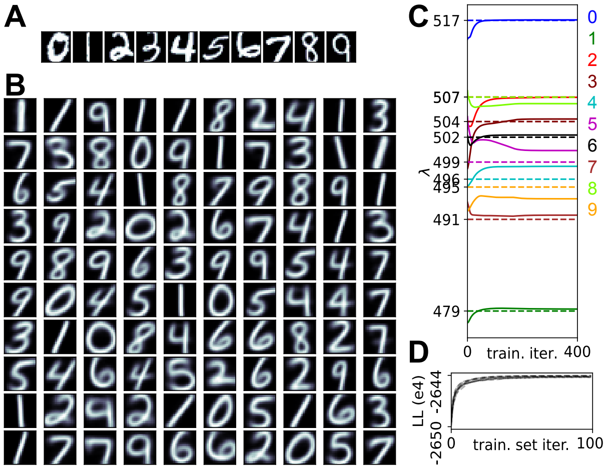

Figure S4. Learning results from the standard MNIST dataset. A: Example data points from MNIST. B: Learned weights for each of the $C = 100$ class units. C: The evolution of the learned intrinsic parameters. Horizontal dashed lines represent the ground-truth values as calculated using labelled data.Different colors correspond to the average of intrinsic parameters of all class neurons that correspond to a given digit, as determined by the Bayesian classifier. D: The evolution of the log-likelihood under our generative model, for ten independent experiments.

Brightness-enhanced MNIST

Fig. S5 presents typical learning results of the model on the brightness-enhanced full MNIST dataset with $C = 20$ class neurons. The mean brightness values were artificially modulated with means sorted in the order of the digit magnitude from the least bright class '0' to the brightest class '9' (see brightness distributions in Fig. S5A). Comparing the exact NB-based posteriors vs. the approximate Poisson solution we see the approximation (Eq. S5) works reasonably well in this dataset. The derived online parameter learning procedure correctly identifies individual digits (Fig. S5C). The estimates of brightness are also usually good, with the exception of digits '4' (light blue) and '9' (orange), potentially because the two classes are difficult to segregate in shape space. Lastly, we show that the learned representation is a good summary statistic of the data. When classifying the digit labels based on the posterior class activities $\mathbf{s}$, performance is improved relative to a solution that uses shape information alone.

S9. Contrastive stress estimation

Finally, we formally define the four methods to estimate the contrastive stress of the sentence presented in Fig. 5 E. Recalling our definition of contrastive stress from the main text:

$$\mathscr{E}(\mathbf{y}) = \langle z \rangle_{\mathrm{P}(z|\mathbf{y},\boldsymbol{\theta})} - \langle \lambda_c \rangle_{\mathrm{P}(c|\mathbf{y},\boldsymbol{\theta})} .$$

Assuming correct and certain classification (i.e. given the label of a stimulus) and inserting Eq. 3 (see main text), we obtain an expression for the Bayes-optimal stress estimate (blue bars, Fig. 5 B, E):

$$\mathscr{E}^B(\mathbf{y}) = \frac{\alpha_c + \hat{y}}{\beta_c + 1} - \frac{\alpha_c}{\beta_c}$$

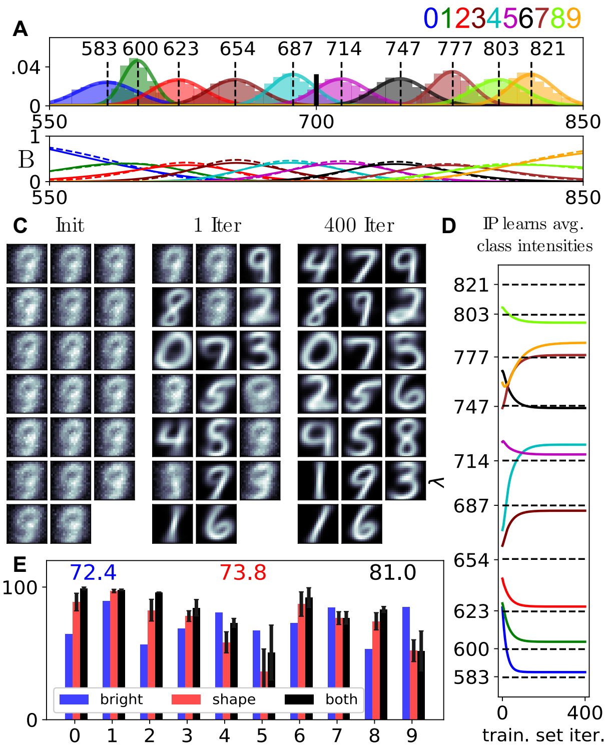

Figure S5. Online learning results for the full, brightness-enhanced MNIST. **A)** Class-dependent brightness histograms after preprocessing MNIST as described in Methods 1 (c.f. Fig. 4 F); Gamma distribution fits in solid lines. **B)** Approx. S5 is reasonably accurate for this dataset. **C,D)** Evolution of synaptic weights (C) and intrinsic parameters (D) during training. Groups of 20 boxes display the full set of weighhts for all C=20 units. The three snapshots of the weights depict the initial conditions (left), after one iteration (middle) and after 400 iterations (right). **E)** Classification performance of the circuit, compared with algorithms that utilize only shape or brightness cues (c.f. Fig. 4 G).

The circuit can approximate $\mathscr{E}^B$ by implementing simple arithmetic computations with quantities that are locally available at each node. Returning to our definition of contrastive stress, inserting Eq. 3 (see main text), $\alpha_c = \lambda_c \beta_c$, and rearranging:

$$\mathscr{E}(\mathbf{y}) = \sum_c s_c \lambda_c \frac{\beta_c}{\beta_c + 1} + \sum_c s_c \hat{y} \frac{1}{\beta_c + 1} - \sum_c s_c \lambda_c.$$

Approximating $\beta_c \approx \beta$ for all c and rearranging, we define the circuit approximation $\mathscr{E}^{IP}$ of $\mathscr{E}^B$ (red bars, Fig. 5 E):

$$\mathscr{E}^B(\mathbf{y}) \approx \mathscr{E}^{IP}(\mathbf{y}) = K(\hat{y} - \sum_c s_c \lambda_c), \tag{S14}$$

where $K \equiv 1/(\beta + 1)$, and s_c depends on $\mathbf{y}$.

Finally, we define two *ad hoc* stress estimates that we propose from intuition. First, let us naively approximate the expected intensity of a stimulus as its brightness, and compare it with the average brightness of all logatomes in the training set. We call this naive stress estimate $\mathscr{E}^N$ (Fig. 5 E, black bars):

$$\mathscr{E}^N(\mathbf{y}) = \hat{y} - \frac{1}{N} \sum_n \hat{y}^{(n)}.$$

Second, we define an improved version of the naive estimate, which we call 'educated naive' $\mathscr{E}^{EN}$ (Fig. 5 E, green bars). We still approximate the expected intensity of a stimulus as its brightness, but now assume access to the ground-truth class-specific average intensities (i.e. the ground-truth λ_c). For the OLLO data we compute each λ_c by averaging the spectrograms of class c which we identify using the OLLO labels. The $\mathscr{E}^{EN}$ estimator is then given by:

$$\mathscr{E}^{EN}(\mathbf{y}) = \hat{y} - \sum_c s_c \lambda_c.$$

Notice that $\mathscr{E}^{EN}$ is proportional to $\mathscr{E}^{IP}$, which we know to be a good approximation to $\mathscr{E}^{B}$. This observation justifies our intuitive definition of $\mathscr{E}^{EN}$ and provides conditions under which the $\mathscr{E}^{EN}$ estimator will be approximately optimal.

While $\mathscr{E}^{IP}$ and $\mathscr{E}^{EN}$ are both accurate approximations of $\mathscr{E}^{B}$, the latter requires (like $\mathscr{E}^{B}$) explicit access to key statistics of the dataset (i.e. the ground-truth λ_c) and it requires a classifier (to compute the s_c). While we have shown that averaging all data points in a class c can provide the required λ_c (see Supplementary sec. 2), such a procedure needs access to the ground-truth label of each data point. Label information (e.g., for each logatome or phoneme) is not available in natural settings, and very costly to obtain in technical applications. In order for the $\mathscr{E}^{EN}$ estimator to be applicable without labels, unsupervised learning procedures and a reliable classifier for new data would be required, which is precisely what the circuit provides via IP. In Fig. 5 E, $\mathscr{E}^{EN}$ (green bars) is calculated using the learned intrinsic parameters of the circuit. However, if we instead calculate $\mathscr{E}^{EN}$ using the ground truth brightnesses calculated directly from the dataset, we see no appreciable difference in the results. This indifference reflects the accuracy of the circuit's learned brightness parameters.

S10. Biological plausible approximations of inference and learning

Here we focus on constructing biologically plausible approximations for the first processing layer, which marginalizes out the unknown input gain z. While the softmax form of inference in our model already has close links to neural circuit dynamics, the interpretation of W_{cd} as the synaptic efficiency of a connection, makes the expression of the current, which depends logarithmically on this quantity difficult to interpret biologically. One potential solution for this issue is to remove the logarithmic dependence of the weights W_{cd} in the current by a further approximation. This can be achieved by rewriting the current to a neuron as $I_c = \sum_d \log(W_{cd}) y_d + \hat{y} \log(\lambda_c) - \lambda_c$, and doing a first order Taylor expansion of $\log(W_{cd})$ around 1, which results in the biologically plausible approximation of the current to a neuron of the form:

$$I_c = \sum_d W_{cd} y_d + \log(\lambda_c)\,\hat{y} - \lambda_c,$$

where one shared term $\hat{y}$ was removed since it cancels out in the subsequent softmax operation. This expression can be interpreted as a traditional weighted sum of linear inputs with a sliding threshold, which now depends somewhat of the overall level of excitation of a neuron's input (either determined internally at the level of the cell, or due to a separate input channel, e.g. via feedforward inhibition, see also main text for interpretation). For the approximation to be accurate, we need the individual weights to be close to 1, which can be achieved by rescaling of the parameters, $\sum_d W_{cd} = A$, with free parameter A chosen to be roughly order D. After rescaling W and z the final expression for the current becomes: $I_c = \sum_d W_{cd} y_d + \log(\lambda_c)\,\hat{y} - A\,\lambda_c$.

The rescaling of variables W, also leads to minor changes in the learning rules (Eq. S8, Eq. S12):

$$\Delta W_{cd} = \varepsilon_W (s_c y_d - s_c \frac{\bar{W}_c}{A} \lambda_c W_{cd}); \qquad \Delta\lambda_c = \varepsilon_\lambda s_c (\hat{y} - A\lambda_c).$$

To quantify the effect of the posterior approximation, we compared the neural circuit solution described above to the abstract circuit dynamics which do not include the approximation of $\log W$. As may have been expected given similar results for a dynamics without intensity inference (Keck *et al*, 2012), the neural circuit correctly recovers the ground truth structure of the training data despite the approximation (Fig. S6). This suggests that approximatively optimal inference and learning in our generative model can be well approximated using neural-like operations.

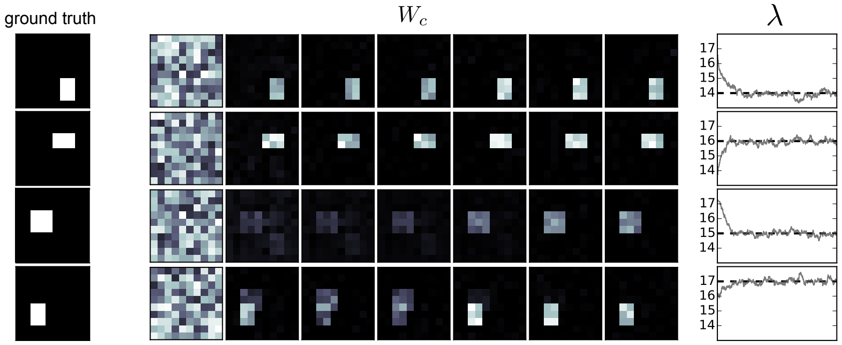

Figure S6. Learning in biologically plausible circuit. See Figs.S1 and S2 for comparison.
